# Souled out of rights? – predicaments in protecting the human spirit in the age of neuromarketing

**DOI:** 10.1186/s40504-019-0095-4

**Published:** 2019-11-22

**Authors:** Alexander Sieber

**Affiliations:** Independent researcher, Brookfield, WI USA

**Keywords:** Human dignity, Human rights, Human spirit, Legal theory, Mental privacy, Neuromarketing, Surveillance

## Abstract

Modern neurotechnologies are rapidly infringing on conventional notions of human dignity and they are challenging what it means to be human. This article is a survey analysis of the future of the digital age, reflecting primarily on the effects of neurotechnology that violate universal human rights to dignity, self-determination, and privacy. In particular, this article focuses on neuromarketing to critically assess potentially negative social ramifications of under-regulated neurotechnological application. Possible solutions are critically evaluated, including the human rights claim to the ‘right to mental privacy’ and the suggestion of a new human right based on spiritual jurisdiction, where the human psyche is a legal space in a substantive legal setting.

## Introduction

Humanity is on the precipice of a technological age that looks poised to irreversibly intrude on the basic human capability of self-determination as well as bodily and mental integrity. Today, technologies exist that can strip the mind of mental privacy. Privacy is a human right protected for all humans under Article 12 of the Universal Declaration of Human Rights (UDHR), Article 17 of the International Covenant on Civil and Political Rights (ICCPR), and elaborated on for the benefit of children in Article 16 of the Convention on the Rights of the Child (CRC), and Article 14 of the International Convention on the Protection of All Migrant Workers and Members of Their Families for those cited. Yet the unique capacities of these new technologies endanger humans in such a way that new ethical measures must be adopted to protect mental integrity. This article examines the problem, views, and it addresses potential solutions to the challenges posed by digital neurotechnology to human dignity.

### A closer look into the Mirror

Since the industrial revolution, humankind in the West has been on a one-way track towards a distinctly Anglo-Saxon vision of development. In the process, untold trillions of US Dollars were invested in capital goods that create even greater wealth by increasing business efficiency. However, today humanity reaps the enormous, compounded detriments known as *opportunity cost*. Ronald Coase’s proverb of opportunity cost – that every transaction has an inherent cost – has become glaringly apparent to modern society. Images of Pacific sea life trapped in plastic trash do not even begin to capture the enormity of the unsustainable development problem, but still, modern society is now somewhat aware of it.

The United Nations has made sustainable development a core ambition in the form of the United Nations Sustainable Development Goals (SDGs). Confronted with the stark reality of industrialization’s unsustainability, the anthropocentric worldview is challenged by a progressive green revolution. Modern society has realized that it shares a biosphere with all living beings. Climate change, displacement, genocide, ethnocide, inequality and lower standards of living have resulted from the pursuit of profit. The conscious effort to reverse the negative consequences of modern development illustrates a paradigm shift: Humankind can ideologically backtrack and innovate with ‘humane’ purpose by working against ignorance as well as corrupt legal and political institutions. This article argues that the application of modern neurotechnology such as functional magnetic resonance imaging (fMRI), electroencephalogram (EEG), and big data should be discussed with the same caution as hydraulic fracking or mass deforestation because all are examples of modern technology posing a significant threat to humanity and all other ecological systems today and even more so if they develop unrestrained. Although the comparison may be shocking to many readers, this paper provides a glimpse into the existential threat humanity faces from the virtually unregulated field of neuromarketing.

### Neuromarketing

fMRI and the EEG have opened new possibilities in neuroscience. Tracking blood flow in brains and reading brain-cell activity, respectively, has led to significant advancements in psychology, but psychology is not limited to the biological sciences. The corporate business world has hailed the brain as the “newest business frontier” (Pradeep and Patel, 2010) In the context of neuromarketing, researchers seek to scrape every bit of information they can from consumers not only to deduce buyer preferences but to decide for the consumer what they shall desire. That is, new media marketing is used to hack and manipulate consumers. Neuromarketers make models based on data collected from, for example, fMRI scans and then market targeted groups based on the likeness to their subjects. It may sound innocent, but deep learning from the marriage between fMRI and big data results in some ominous futures.

For example, Google, LLC has made neuromarketing part of its marketing strategy. Looking to tie its product to the subconscious of its advertisement viewers, Google’s scheme relies on human psychological attachment to its advertisement characters to obtain the attention of the viewer. In such a presentation, neuromarketing is fundamental. Just as fast-food giants use colors that induce hunger in their restaurants for monetary gain, Google uses primal and emotional triggers of the human unconscious to generate business.

Today, companies like Google, Samsung, and Facebook engage in extremely invasive and non-transparent neuromarketing strategies and techniques often in the name of efficiency. For example, Facebook, Inc. is engineering neurotechnology that transfers user thought to text. Allegedly, this technology is to save the consumer’s time. This invasive methodology marks a new rupture in marketing that could have only been made possible with the advancement of technology.

Currently, neuromarketing mines hidden information and even manipulates the consumer’s mind by accessing brain information. The mind was once presumed to be a kind of haven from intrusion from the outside world. This is no longer true in modern capitalist society. The mind has been tapped, but what if a line is not drawn at the consumer; what if neuromarketing becomes the method by which a state controls groups? Philosopher Michel Foucault feared this. In his writings on *biopolitics*, Foucault provided a genealogy of and predictions for the application of power over living bodies and whole populations. Before dying in 1984, Foucault critiqued neoliberalism. Today, Neo-Foucauldians continue his analysis of neoliberalism as a manifestation that has given rise to greater exploitation, inequality, and corruption of individuals and their highest ideals, such as human dignity and democracy (Brown 2015).

### How we got Here

Neoliberalism has created tremendous wealth in the digital age. Austerity measures propel businesses towards greater innovation such as neuromarketing. The financial stakes are so high now that humankind is mining the mind to find out what product is preferred, but that is just the beginning. Neuromarketing now aims to spread and alter specific brain data and brain functions – amounting to consumer manipulation. As Nikolas Rose explains, the “re-shaping of human beings is thus occurring within a new political economy of life in which, in part at least, biopolitics has become bioeconomics (Rose 2007).” The link between contemporary capitalism and efforts to strip the mind is unmistakably evident. Modern capitalism has explicitly brought forth dehumanization and objectification.

Reducing a human being to a thing devoid of agency and self-determination degrades the human by othering it. As the person becomes an object, it loses its dignity. Under an fMRI scanner, the human brain is a ‘purchaser’ and nothing more to the neuromarketer. The genealogy of mind-raiding indicates that the next step is to trigger specific action from the objectified purchaser. As Selena Nemorin writes:

In their deliberate efforts to trigger and condition consumers into buying responses, neuromarketing practitioners actively seek to manipulate human processes of understanding, which results in the subversion of core democratic values of freedom of intelligence and self-determination (Nemorin 2018).

In effect, neuromarketing could undermine democracy, as the foundation of civil society is to have rational people making decisions on their community’s and their own behalf. By definition, rationality is a calm, calculated state. If one takes away the human’s communal standing and rationality, one is left not merely with a homo oeconomicus (for even that would require rationality) that acts rationally in its market decisions but with a *homo alalus*: a machine-like man.

It is perfectly acceptable to treat machines as machines and animals as animals, but, as European Enlightenment philosopher Immanuel Kant’s categorical imperative tells us, humans are to be treated not as a thing, but as an end in itself. Humans are different from other creatures because they are born with the self-determination to be moral and thus to enshrine with dignity. Without autonomy, dignity is lost. *Homo sapiens*, the moral animals, that have been othered to the point of being treated as a laboratory experiment subject that has not been given free, prior and informed consent (FPIC) – like a regular laboratory animal – lose all dignity. Self-determination, dignity, and FPIC are protected as universal human rights. Rights are designed to protect the dignity of humanity – thought to be the essence of being a human.

Without dignity, humans and their societies disintegrate because humans are creatures of profound ethical choice and power over their environment, including over their peers as they all are being-towards-death and therefore, command meaning. In a life of phenomenon, expresses Gail Linsenbard: “ontological freedom, or freedom of choice… is foundational in that it is what all persons are as Being – as human reality, and it makes practical freedom (or unfreedom) possible (Linsenbard 1999).” It is through freedom to love and play that meaning is generated in society. Love is only generated when individuals have a ‘world’ as described by Hegelian philosophy. Psyches guide each of our worlds, and we can, of course, share – thus further our loving, though without meaning there is nothing for the *Homo sapiens*.

Then there is world destruction. The exhumed psyche is a shattered world. Few things can be conceived that match it. Phenomenologists have long been interested in world destruction. Examples include killing someone and catastrophic loss, but few of us know what experiencing mind mining is really like. Violation of what Nemorin calls “boundary-integrity”^1^ happens when minds are hacked. Nemorin’s use of the word ‘boundary’ suggests a value in maintaining a *profanum* – where the sacred and the profane dichotomy is definite – suggesting that law is the answer.

#### Social consequences of Neuromarketing

Laissez-faire policies created incentives for modern society’s re-enslavement; they also render the society paradoxical, as there is nothing liberal about living in a community that is devoid of choice, even if it is so in the name of ‘development’. As consumers have their minds tapped, and corporations hurtle humankind towards a neuromarketing dystopia, one has to ask: How does class fit into the futureless age, and would neuromarketing become a tool of the bourgeoisie to suppress the market consumer who will never hold power over a corporate board? Additional to the horrors of bioeconomics are humankind’s trajectorial vestiges of what Marxist philosopher Antonio Gramsci called ‘cultural hegemony’. A world in where neuromarketing runs amok may well lead to the awful realization of a global class society. Giving in to extreme or unencumbered neuromarketing is giving in to the norm of having a ruling class. There is no way around the personal neuromarketing problem other than to rule it. Should humans let neuromarketing strip them bare of their human rights, or is there a different route?

It is important to note that neuromarketers “do not actively *see* the consumer as an animal,” but the neuromarketing industry “maintains a conceptual reduction of the consumer to [the] brain as animality is re-inscribed through the discourse, assumptions and operative practices and goals.”^2^ Othering can render policy discourse about consumers faulty on how marketing is helpful, and consumers are rational. For the sake of ethical argumentation, laissez-faire policymakers should refrain from belittling neuromarketing’s nefarious side as many historically have done in debates on the human involvement in climate change.

### Futures in human rights

I have put forth the idea that “[Hu]mankind must declare a normative space to contemplate what it means to be human, for there is a necessity and [a] value to the spiritual as a space in which we can conceptualize the human spirit that is not subject to Empire (Sieber 2017).” This idea of jurisdiction may be a solution to the problems posed by neuromarketing as well as to other attacks on the human spirit, but it raises some interesting issues regarding governmentality.

Human rights have always had a metaphysical level to them. Protected in the Preamble to the UDHR is a reference to “inherent dignity (UN General Assembly 1948).” Humanism has a spiritual streak, as features of sacred and profane are fundamental to the human rights regime. Laws of sacredness and profanity are laws observed and enforced today: Freedom is sacred and therefore protected by law, while bodily harm is profane and prevented by law. Humans are owed a level of privacy and space that, again, are protected by universal human rights. Today, our privacy bubbles are being hacked: what was once assumed to be sacred is now profane.

Jurisprudential language regarding persons relies heavily on the Ancient Greek, Ancient Roman, and Christian understandings of what they considered the real person and the *persona*. The persona is the mask a real person wears (Burchell 1998). A person may wear his or her dignity and rights as a persona where the mask is something constructed as purely legal. What I called for was a return to the old notion of spiritual jurisdiction. Jurisdiction is a technology that has a toolkit that includes categories. Categorization of the psyche as a legal space would mark a return to substantial law – not to assume, but to assert rights.

What potential adversity could this measure produce? Entrenched in today’s rights societies is something that Costas Douzinas calls “Foucault’s law”: The situation in where postmodern societies feel more insecure and unfree the more rights are had (Douzinas 2007). In other words: Rights have diminishing returns – especially nuanced rights – because they frame rights-holders in some way or another. Take for example the Convention on the Elimination of All Forms of Discrimination Against Women (UN General Assembly 1979). Women’s rights are important for ensuring the dignity of women, but they still frame the individual woman by her ‘gender’. Now, consider how having a legal space for the psyche in the context of human rights would frame and be received by rights-holders. Not everyone may embrace such an invasive, indeterminate, aspirational human right that possesses a seemingly antiquated premise; they may not want to venture into the governmentality involved in overseeing – of all things – the human soul.

A further caveat to my proposition is the law and economics case of diminishing returns and the quest for efficiency. International human rights, which have been subjected to new zealous – albeit deficient – critiques by law and economics scholars(Posner 2014) may continue to use utilitarian arguments to justify challenging human rights in general. Law and economics scholars may make a legitimate case against the jurisdiction of the psyche on the basis of efficiency, but where does that leave humankind? It leaves humankind with a problem that perhaps cannot be solved (the jurisdiction of the human psyche), or – in the name of pragmatism – should not be addressed. Therefore, perhaps the most viable solution to the neuromarketing question remains to police individual applications and technologies – however more jumbled that may prove to be legally.

### “The flame”

There is a significant precedent in maintaining protection for what one might call *the flame* or the human spirit. Albert Einstein once said, “The human spirit must prevail over technology.” In the age of unrestrained neuromarketing, one has reason to believe that technologies can intentionally or unintentionally extinguish the human spirit. Neuromarketing and other technologies can obliterate culture and meaning. The arts, which are greatest when agents are introspective, suffer the most in a neoliberal society where they are cut off from reality by neuromarketing and virtual existence. The arts are avenues for spiritual power that keeps the flame burning.

Philosopher Martin Heidegger was notoriously skeptical of technology. He thought that *enframing* reduces imminence between individuals in society by isolating them. Art, he argued, destroys barriers and brings people closer to truth and authenticity. Heidegger wrote in 1954 that “Enframing means that way of revealing that holds sway in the essence of modern technology and that it is itself not technological (Heidegger 1977).” Furthermore, “the rule of enframing threatens man with the possibility that it could be denied to him to enter into a more original revealing and hence to experience the call of a more primal truth.”^3^ That is, truth escapes humankind unless it tames or turns away from the monstrous apparati it has made. It is Art – the very subject that modern technology suppresses – which acts as a beacon in dark times such as contemporary capitalism. Truth can be difficult or even impossible to grasp when communication is stifled because understanding is subsequently rendered foggy and dumbed-down. Under an unfettered, dystopian neuromarketing regime desire for truth and meaning go down the drain, as society irreversibly become post-truth, post-reason, post-culture, post-meaning, and post-freedom, as the arts are reduced to paradoxically insipid sensationalist tabloids.

### Psychopolitics

Foucault wrote in 1975 that “the soul is the prison of the body (Foucault 2012).” Just as biopolitics is the politics of bodies, *psychopolitics* are the politics of the psyche or ‘soul’. Psychopolitics has been synonymous with torture because the first book published on Soviet torture techniques was titled *Brain-Washing: A Synthesis of the Russian Textbook on Psychopolitics*. Unlike Foucault’s biopolitical systems of coercion, modern psychopolitics represses and seduces the human psyche. As sinister as psychopolitics sounds and as futile the existence it renders sounds, they are part of the new status quo.

In *Psychopolitics: Neoliberalism and New Technologies of Power* philosopher Byung-Chul Han bridges the idea of biopolitics of coercion and violence. Violence is “the intentional use of physical force or power, threatened or actual, against oneself, another person, or against a group or community, which either results in or has a high likelihood of resulting in injury, death, psychological harm, maldevelopment, or deprivation (Krug et al. 2002).” Big data and modern neurotechnology have the potential to be violent because of their use of psychopolitics on the human psyche. In his book,

Han argues that digital psychopolitics can intervene in human psychic processes. One of his worries about psychopolitics’ microphysics of power is that it will be even faster than human free will – meaning that, when left unchecked, psychopolitics may very well beckon the end of human freedom. Humans would no longer be able to subdue big data’s microphysics because of the microphysical dimension it operates in (Sieber 2019).

Freedom is protected by Article 1 of the UDHR, but if the human mind can no longer subdue big data’s microphysics because of the dimension it operates in, then how can humanity cope with the dangerously unmanaged marriage between big data and neurotechnology? As promising as digital technology may be, a more in-depth look shows that “when left unaccounted for, it offers no future or reason yet remains commander of humanity’s future.”^4^ Therefore, it must become a human sustainability project to address neuromarketing.

### Mental privacy as a human right

Many bioethicists have promoted discussion on neurospecific rights. The advocates of implementing mental privacy as a human right say, “the neurotechnological future we are approaching will require us to guarantee protection not only to the information we record and share, but also to the source of that information since they may be inseparable (Ienca and Andorno 2017).”^5^ But what would enforcing mental privacy as a human right entail, and in what meaningful way can a duty-bearer protect rights bearers from a pernicious process that they are oblivious to? Researchers Marcello Ienca and Roberto Andorno propose “the formal recognition of a right to mental privacy, which aims to protect any bit or set of brain information about an individual recorded by a neurodevice and shared across the digital ecosystem.”^6^ They are adamant that humans “need wider privacy and data protection rights” and that “the need to protect information generated below the threshold of voluntary control demands for the recognition of a new right that is specifically tailored on the characteristics of brain information and the new possibilities opened by mind-reading technologies,”^7^ yet fail to address *how* that should be done. One can always write rights, but one cannot necessarily protect those rights.

Governmentality entails that the people trust the state with the morals of its government. Moreover, governmentality shapes the practices that “try to shape, sculpt, mobilize and work through the choices, desires, aspirations, needs, wants and lifestyles of individuals and groups.”^8^ An intensely moral agreement between the people and its leadership, governmentality has the power for good or evil: benign purpose or invidious purpose. Protecting citizens’ rights is often the initial intent, yet rights can fail in the form of re-enslavement. Worse yet, data collection – which is inherent to governmentality – could be used by a regime to abuse groups. A regime of human rights is not guaranteed to be a regime of peace.

#### Regulation of Neuromarketing

As Han writes in his book *Topology of Violence*, “Regulation is always accomplished as spatialization and localization. Sheer violence alone is not capable of forming spaces or creating location. It lacks the space-building force of mediation. Thus it cannot produce a legal *space*.”^9^ In an example, Han makes note that power always involves consent, as “Power, in contrast, develops along a yes.”^10^ He writes, “The greater the popular approval of the ruler, the greatest is the ruler’s power.”^11^ He reiterates, “Even forcible subjugation contains a yes.”^12^ Absolute violence is fundamentally different from power in this sense.

Han contends that “An absolute no negates the relationship of power, that is, subjugation.”^13^ Furthermore, “Contemporary violence is based more on the *conformity of consensus* than the antagonism of dissent. Thus one could invert Habermas’s phrase and speak of the *violence of consensus*.”^14^ Han ends his dismantling of expanding legal spaces to detect violence with a blaring analysis of the new status quo: “Today, politics itself is positivizing itself into work without any possibility of *sovereign action*.”^15^ This means that not only would it be nearly impossible to identify any evidence of neuromarketing’s violent psychopolitical actions, but the conception of a soular jurisdiction (i.e., a jurisdiction of the human psyche) is also rendered futile. Neoliberalism’s subtle, stealthy manifestation known as ‘neuromarketing’ can no longer be traced or brought to justice, so the only conceivable way of curbing its negative effects is through regulation of the devices themselves. Insofar, my proposition of a jurisdiction or legal space for the contemplation on the human spirit may well be necessary, but it will not suffice in actually protecting the human psyche from violence.

### Alternative futures

Heidegger famously said in an interview: “only a god can save us.”^16^ He was referring to the kind of hegemonic predicaments humankind is in now. To put Heidegger in context, we must look to the law. In natural law, any end is justified, yet in positive law, any end is justified by its means of creating order. Philosopher Walter Benjamin made note of *divine violence* as violence that maintains law and order. With divine violence, there are no ends but a call from within.^17^ It is called ‘divine’ because it is outside the flow of history and time; it is a pure manifestation that is both spiritual and apostate. Civil disobedience, on the other hand, is peaceful political protest that may be impossible under “neuroscientific and neurotechnological imperialism.”^18^ Heidegger meant that, if anything, the holy (not God itself) could save humankind from its demise. Philosophy will not solve the problems of modernity, but meditation that leads to a new way of thinking may. Hence, only a god can save us, says Heidegger. Although convexly worded, I think Heidegger would consider human rights as legal technology and the ideal of human dignity a poetic necessity. Insofar, human rights could continue their role as humanity’s redemptive scaffolds, or law could prompt the necessary reengineering of technology to comply with our uniquely human needs.

Another alternative to positive law that renders a field whereby one can interpret mental privacy is to accept a heretical existence. Society may have to settle for the future it is getting. If so, humankind may learn to redefine the individual body and self. Human agency is manifold. One must then ask: Why would one oppose losing a fraction of agency to neuromarketing? Humans are born unfree; they live their entire lives without *total* free will; they enslave themselves to laws, yet nevertheless, they live. What is stopping people from voluntarily losing more agency to technology if it is economically prudent? Jurisdiction would open the door to debate on the right to property. Much like selling one’s own organs on an open market, may voluntarily selling access to one’s mental privacy be made legal? This law and economics course would cut human rights to its groundless core – an angst-reducing and a sanctity-creating mechanism that is altogether disinterested in the unknown and functions primarily in absolutes in a world that may command a retreat therefrom.

Then remains the rather profitable, albeit still realistic, future of a commercial good produced that can be worn as a physical persona. Fitted like a mask, humans around the world who choose to opt-out of surveillance (and can afford to) could wear a concealment or use a disorienting mechanism (e.g., surgical masks, digital-camouflage garb, makeup, laser pens), as we have seen in the recent Hong Kong resistance in the heavily surveyed Chinese metropolis. This would likely pose a mere blip for big data collection, as it will likely amass enough information to compensate. In such a case, the persona would be a cost-bearing item, possibly furthering any economic divide.

Lastly, technology is inherently innovative. This is true for efficiency and for correcting economic externalities of production. Take the green revolution for example. Humanity found ways to generate energy while curbing carbon dioxide emissions. There, it innovated to create similar production outputs, if not more and reduced emissions. With nuclear arms, humankind has largely chosen to deescalate and scale down, but one can never *uninvent* the bomb. Any effort to enforce ignorance about the brain in marketing or data collection would be what economists call a *market distortion*: insufficient supply for the quantity demanded because of interference. In the past, these have led to criminal activity (e.g., the trade of drugs and people). Furthermore, data collectors may not be retroactively stripped of the information they legally obtain. How data is managed from now on is an issue in itself.

## Conclusions

This article examines the implications of new technologies that encroach on a space (the psyche) alleged to be sacred. In such a context, it approached the topic as an ethical issue by evaluating the implications of escalating legal measures and also the absence thereof.

If “the modern notion of *human* dignity involves an upwards equalization of rank, so that we now try to accord to every human being something of the dignity, rank, and expectation of respect that was formerly accorded to nobility,”^19^ then, there is no way for neurotechnological imperialism to progress in a way that does not increase the gulf between the wealthy class and the working class if it will be an instrument for cultural hegemony. In that respect, the digital revolution’s dystopic course is socially damaging to the human value of dignity.

Furthermore, the use of neurotechnology can violate citizens’ universal human rights to self-determination and privacy that may never be regained given the dystopic trajectory. The number of voices calling for new human rights is growing, and they are seemingly legitimate. That being said, theorists allege that this may be a lost cause since it is unrealistic to govern the psyche in any pertinent sense. The human right to mental privacy remains aspirational and has gained some traction. Ienca and Andorno’s paper on what they call a ‘right to mental privacy’ recommends new regulation that may be the only answer to the dangers of unfettered neurotechnology.

If humankind lives in an age where modern technology has pressed it to react to technology for the sake of human dignity, we have reached truly a historic point in history. No longer are we in the state of nature where conflict exists solely between humans, Earth, and animals. Now humankind’s creations (technology) have created new points of conflict. The Hegelian master–slave dialectic is perhaps better understood today as, say, the human–computer dialectic. Both fight each other for autonomy – the only difference is that stealthy neurotechnology depends on an apathetic, willing human population. These issues are what make the discourse on neurospecific rights and regulation so remarkable.

Neurospecific rights would also contribute to the reduction in the sleek glamour of the still relatively young international human rights law regime. The law and the concept of human dignity have been made messy by technology. This stands to remind scholars that the law itself is technological – an evolving human construct.

Lawcraft that pushes back against allowing technology designed to use human faculties as a resource at the expense of individual human dignity should be cautiously embraced. In a neoliberal age, regulation is a necessary yet not so sexy political stance. Civil society should be informed about the dangers posed by neuromarketing before giving in to its soft hegemony. Equal-standing citizens deciding with clear eyes on what is just is part of sufficient democracy. But legal scholars cannot forget that law itself is technological, and insofar, the concept of human dignity and themes surrounding it (e.g., freedom, conditions for valid consent) must be further expounded upon.

Neuromarketing and other modern neurotechnologies that harness the power of the human psyche will challenge what it means to be human because it is not clear that there will be a universal response to its harmfulness. A capitalist agenda may triumph – making citizenry bear the economic cost of maintaining a literal persona.

Finally, this article questions the efficiency (i.e., payoff) of intrusive neurotechnology. If there is a net benefit, there is – albeit a small – space in legal scholarship for condoning pernicious technology. The time is now for people to debate and set a legal precedent. The applications of big data and neurotechnology deserve the attention of activists, ethicists, lawyers, and policymakers. Neuromarketing, in particular, has enjoyed life on a lawless frontier and should be subjected to delibrative governing before it fully mutates into a digital-aged form of *barbarism*: when technology (neuromarketing), spirituality (worship of the Market), and the politics of custom (mind-reading) are used to commit acts of extremely cruel conquest (cultural hegemony in the creation of a subjugated class), where the whole are promised salvation (greater efficiency) and the few (dissenting voices) are cast aside for their infamy.

## Data Availability

Not applicable

## Abbreviations

CRC: Convention on the Rights of the Child
EEG: Electroencephalogram
fMRI: Functional magnetic resonance imaging
FPIC: Free, prior and informed consent
ICCPR: International Covenant on Civil and Political Rights
SDGs: Sustainable Development Goals
UDHR: Universal Declaration of Human Rights

## Footnotes

1 Nemorin, *Biosurveillance in New Media Marketing*, 213.

2 Ibid., 216.

3 Ibid.

4 Sieber. “Does Facebook Violate Its Users’ Basic Human Rights?,” 143.

5 Marcello Ienca and Roberto Andorno, “Towards new human rights in the age of neuroscience and neurotechnology.” *Life Sciences, Society and Policy* 13.1 (2017): 14.

6 Ienca and Andorno, “Towards new human rights in the age of neuroscience and neurotechnology,” 15.

7 Ibid., 14.

8 Mitchell Dean, *Governmentality: Power and Rule in Modern Society*, Second Edition (Washington: Sage, 2010), 20.

9 Byung-Chul Han, *Topology of Violence* (Cambridge: MIT Press, 2018), 56.

10 Han, *Topology of Violence*, 56.

11 Ibid.

12 Ibid., 57.

13 Ibid.

14 Ibid., 62.

15 Ibid.

16 *See* Martin Heidegger, “Nur noch ein Gott kann uns retten,” trans. W. Richardson *Der Spiegel* 30 (May, 1976): 193–219.

17 *See* Walter Benjamin, *Zur Kritik der Gewalt und andere Aufsätze* (Frankfurt: Suhrkamp Verlag, 1921)

18 Andrea Lavazza, “Freedom of thought and mental integrity: The moral requirements for any neural prosthesis.” *Frontiers in Neuroscience* 12 (2018): 84.

19 Jeremy Waldron, *Dignity, Rank, and Rights* (New York: Oxford University Press, 2012), 33.
